# Maximum inspiratory pressure, a surrogate parameter for the assessment of ICU-acquired weakness

**DOI:** 10.1186/1471-2253-11-14

**Published:** 2011-06-26

**Authors:** Georgios Tzanis, Ioannis Vasileiadis, Dimitrios Zervakis, Eleftherios Karatzanos, Stavros Dimopoulos, Theodore Pitsolis, Elli Tripodaki, Vasiliki Gerovasili, Christina Routsi, Serafim Nanas

**Affiliations:** 1 First Critical Care Department, Evangelismos Hospital, National and Kapodestrian University of Athens, Athens, Greece

## Abstract

**Background:**

Physical examination has been advocated as a primary determinant of ICU-acquired weakness (ICU-AW). The purpose of the study is to investigate ICU-AW development by using Maximum Inspiratory Pressure (MIP) as a surrogate parameter of the standardized method to evaluate patients' peripheral muscle strength.

**Methods:**

Seventy-four patients were recruited in the study and prospectively evaluated in a multidisciplinary university ICU towards the appearance of ICU-AW. APACHE II admission score was 16 ± 6 and ICU stay 26 ± 18 days. ICU-AW was diagnosed with the Medical Research Council (MRC) scale for the clinical evaluation of muscle strength. MIP was measured using the unidirectional valve method, independently of the patients' ability to cooperate.

**Results:**

A significant correlation was found between MIP and MRC (r = 0.68, p < 0.001). Patients that developed ICU-AW (MRC<48) had a longer weaning period compared to non ICU-AW patients (12 ± 14 versus 2 ± 3 days, p < 0.01). A cut-off point of 36 cmH_2_O for MIP was defined by ROC curve analysis for ICU-AW diagnosis (88% sensitivity,76% specificity). Patients with MIP below the cut-off point of 36 cmH_2_O had a significant greater weaning period (10 ± 14 versus 3 ± 3 days, p = 0.004) also shown by Kaplan-Meier analysis (log-rank:8.2;p = 0.004).

**Conclusions:**

MIP estimated using the unidirectional valve method may be a potential surrogate parameter for the assessment of muscle strength compromise, useful for the early detection of ICU-AW.

## Background

ICU-acquired weakness (ICU-AW) is a well recognised complication, with an incidence of 10-25%, among long-term mechanically-ventilated patients [1-5]. ICU-AW presents with profound muscle weakness and difficulty to wean from mechanical ventilation (MV); it results in prolonged Intensive Care Unit (ICU) and hospital stay and even in increased mortality [6,7]. Clinical, electrophysiological and histological approaches have been used for the diagnosis [2,8,9]. Clinical detection is based on the assessment of skeletal muscle weakness. A standardized and reliable method to evaluate limb muscle groups is the Medical Research Council (MRC) muscle strength score [10-12] which has been used for the diagnosis of ICU-AW [2,9,13-15].

Although ICU-AW was firstly described in patients with prolonged time to successful weaning from the ventilator [16], specific estimation of respiratory muscle strength reduction in the context of this neuromuscular disorder has been addressed only in a recent study by De Jonghe et al [17]. This study confirmed the association between respiratory muscle and skeletal muscle weakness using the standard methodology of measuring respiratory pressures.

Previous studies have shown that systemic diseases with peripheral muscle involvement also affect the respiratory muscles, reducing the pressures developed during respiration [18].

The clinical detection of ICU-AW by MRC muscle strength score appraisal as well as fully informative study of respiratory dynamics presupposes that patients have sufficient level of consciousness to cooperate and respond to orders [10,11]. A method to assess muscle strength in patients that are not yet able to cooperate after critical illness recovery has not been described so far. Furthermore, the time required for the patient to regain consciousness seems nevertheless precious in terms of possible early beneficial precautions and interventions that could lessen the probability of developing clinically significant neuromyopathy.

Our hypothesis was that ICU-AW could equally affect respiratory muscles, as the peripheral muscles of the limbs, resulting in reduced respiratory muscle strength and diminished inspiratory pressure developed during the respiratory efforts. Assessment of respiratory muscle strength in the critically ill patients before they fully recover could be an alternative method for the early detection of ICU-AW.

The aim of this study is to investigate a surrogate index of ICU-AW by estimating inspiratory muscle strength with the unidirectional valve technique that can be applied in ICU patients without the need of full cooperation.

## Methods

### Patients

The study was approved by the Scientific Council and the Ethics Committee of our Hospital. Family members of all patients that were included in the study gave written informed consent.

Seventy-four patients with ICU stay ≥ 7 days, were recruited in the study and prospectively evaluated in a multidisciplinary university ICU towards the appearance of ICU-AW. Exclusion criteria were age < 18 yrs, presence of pregnancy, limb fractures, body-mass index > 35 kg/m^2 ^and known pre-existing causes of neuromuscular weakness. Patients characteristics are presented in Table 1.

**Table 1:** Characteristics of critically ill patients evaluated for inspiratory muscle strength that enrolled in the study (N = 74) and of the above critically ill patients that MRC muscle strength score could be estimated (N = 33)

Variables	N = 74	N = 33
Age, years	59 ± 20	55 ± 20
Gender, Male/Female	52/22	29/4
COPD	10	5
Chronic heart failure	4	3
Diabetes mellitus	11	4
Medical/Surgical/Trauma	26/27/21	8/14/11
Main diagnosis		
Pneumonia	6	1
Sepsis	5	2
COPD	1	1
Trauma/Burn	21	10
Cerebral hemorrhage/surgery	14	4
Cardiothoracic/Vascular surgery	4	2
Abdominal surgery	10	7
Drug intoxication	1	1
Other	12	5
ICU Stay, days	33 ± 17	32 ± 18
APACHE II score	16 ± 5	15 ± 5
SOFA score	8 ± 3	7 ± 3
SAPS III score	57 ± 14	55 ± 12
ICU-AW/no ICU-AW		17/16
MRC muscle strength score, (n = 33)		41 ± 16
MIP, cmH_2_O	37 ± 18	38 ± 17
Length of MV until onset of weaning, days, (n = 66/31)	16 ± 13	13 ± 12
Weaning period, days, (n = 60/30)	6 ± 10	7 ± 11

COPD = Chronic obstructive pulmonary disease, ICU = Intensive Care Unit, APACHE = Acute Physiology and Chronic Health Evaluation, SOFA = Sequential Organ Failure Assessment, SAPS = Simplified Acute Physiology, ICU-AW = ICU acquired weakness, MRC = Medical Research Council, MIP = Maximum inspiratory pressure, MV = Mechanical ventilation.

All continuous variables are presented as mean ± SD

### Design of the study

All patients that fulfilled the inclusion criteria were thoroughly followed-up during the ICU stay. Assessment of illness severity on admission was performed by the APACHE II, SOFA and SAPS II prognostic scores [19-21]. Trials of weaning from the ventilatory support were started after daily clinicians' decision according to the patient's clinical conditions. All patients underwent an assessment of respiratory muscle strength by measurement of maximum inspiratory pressure (MIP) after a 48 hours discontinuation of sedation and MRC muscle strength scale when patients regained consciousness, if possible within 48 hours from MIP measurements.

### Maximum inspiratory pressure

MIP was measured with the method described by Marini et al [22] where a unidirectional expiratory valve is used to selectively permit exhalation while inspiration is blocked. One side of the valve was attached to the patient's endotracheal tube and the other side to a manometer. Manometer could register pressures from 0 to 100 cmH_2_0. Patients were disconnected from the ventilator and were attached to the valve for a period of more than 30 sec and the maximal value was recorded. Three maneuvers were performed and the highest value was used for the analysis. Conduction of this process does not require the coordination of the patient whereas it permits MIP assessment at lung volumes progressively closer to residual volume. In an effort to allow respiratory drive reappearance, MIP assessment was performed approximately 48 hours after the discontinuation of sedation.

### MRC muscle strength scale

The patients were assessed on a regular basis for the development of neuromuscular weakness. The MRC scale for clinical assessment of muscle strength [10-12] was used for the diagnosis of ICU-AW with values ranging from 0 (quadriplegia) to 60 (normal muscle strength) and with a score of 48 being the cut off point for the diagnosis of ICU-AW. We adopted a cut off limit of 48 for the MRC score because it reflects clinically significant weakness and has been used previously for the clinical identification of ICU-acquired paresis [2]. MRC score was performed by two independent investigators that were familiar with this technique. MRC was obtained on the day the patients had level of consciousness adequate to respond to at least three of these orders ("open/close your eyes", "look at me", "put out your tongue", "nod your head", "raise your eyebrows").

### Weaning from mechanical ventilation

T-piece trial was used as a screening method for weaning after daily clinical evaluation. Weaning period was defined as the time from the onset of weaning trials until the day there was no need for MV for the next 48 hours. Weaning was considered successful when no ventilatory support was necessary for at least two days. The duration of MV, the duration of onset of MV to onset of weaning and the duration of weaning period were all recorded.

### Statistical analysis

All continuous variables are presented by mean ± SD. Group means of continuous variables were compared by unpaired Student's t-test. The association between MIP and MRC muscle strength score was analyzed by Pearson's correlation coefficient. The cut-off value for MIP to predict ICU-AW was based on receiver operating characteristic (ROC) curve analysis. The Kaplan-Meier method was used to compare the duration of weaning between patients with and without ICU-AW and to compare weaning period between patients that had MIP below or above the cut-off value. We compared the two groups using the log-rank test. The lowest level for statistical significance was set at p < 0.05.

## Results

Patients' characteristics enrolled in the study are reported in Table 1. From the 74 patients, 8 patients died without reaching the weaning period and 5 patients died after weaning process. One patient was transferred to another ICU after a long period of weaning trials. Sixty patients were successfully weaned from the ventilator.

The mean value of MIP in the critically ill patients (N = 74) tested was 37 ± 18 cmH_2_O. Among the 74 patients enrolled in the study 39 patients did not have a sufficient level of consciousness for the assessment of limb muscle strength by the MRC score and the diagnosis of ICU-AW could not be established. This group consisted of 14 patients that died and 25 patients that never regained a sufficient level of consciousness until the discharge from the ICU. Two patients were overlooked and MRC was not estimated. Consequently MRC was safely estimated in 33 patients and its mean value was 38 ± 17. The mean time difference between MIP and MRC testing was 5 ± 5 days. Characteristics of patients with MRC muscle strength score evaluation are presented in Table 1. From these patients, three patients died before weaning period started and 30 patients were successfully weaned from mechanical ventilation.

**Figure 1:**
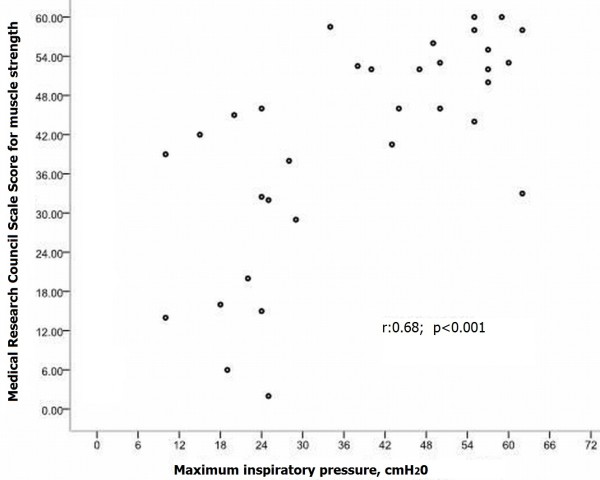
**Scattergram of maximum inspiratory pressure and medical research council scale for muscle strength in critical ill patients enrolled in the study, (r:0.68; p < 0.001, N = 33)**.

A significant correlation between MIP and MRC muscle strength score was found in the core material of 33 patients that were amenable to both measurements (r = 0.68, p < 0.001, Figure 1).

Patients that developed clinically ICU-AW (51%) had a longer ICU stay (41 ± 19 versus 21 ± 11, days, p = 0.001), a lower MIP (28 ± 15 versus 49 ± 11, cmH_2_O, p < 0.001), and a longer MV weaning period (12 ± 14 versus 2 ± 3, days, p < 0.05).

The value of 36 cmH_2_O for MIP was found as the best cut-off value for the diagnosis of ICU-AW from the ROC curve analysis [AUC (area under the curve) = 0.85; CI:0.71-0.99, p = 0.001)]. The value of 36 cmH_2_O had a sensitivity of 88% and a specificity of 76% for the diagnosis of ICU-AW.

In a prospective analysis of the 74 patients included in the study, patients with MIP < 36 cmH_2_O were older (67 ± 16 versus 51 ± 7, years, p = 0.001), had a more severe APACHE II and SAPS III score [(17 ± 4 versus 15 ± 5, p = 0.05) and (62 ± 14 versus 53 ± 12, p = 0.05), respectively], a longer ICU stay (37 ± 6 versus 28 ± 13, days, p = 0.001), and a longer MV weaning period (10 ± 14 versus 3 ± 3, days, p < 0.01).

### Kaplan-Meier analysis

**Figure 2:**
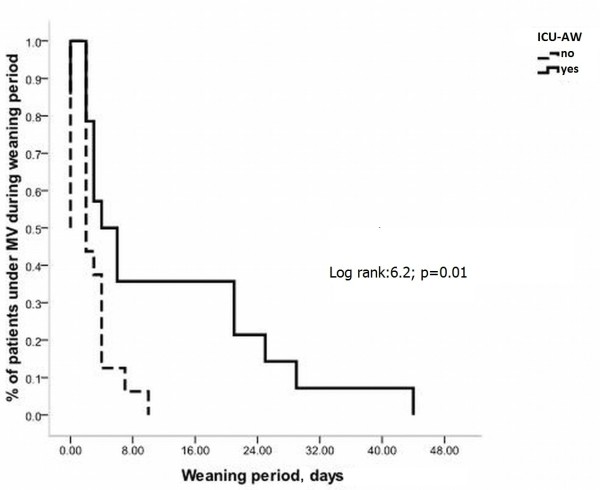
**Kaplan-Meier curves of the probability of remaining under mechanical ventilation (MV) after the onset of weaning according to the presence or not of ICU-acquired weakness (ICU-AW)**. A cut off limit of 48, for the medical research council scale (MRC) muscle strength score, has been adopted for the diagnosis of ICU-AW.

Patients with ICU-AW had a greater daily probability of remaining under mechanical ventilation (MV) after the onset of weaning compared with those patients without ICU-AW (log-rank:6.2; p = 0.01, Figure 2).

**Figure 3:**
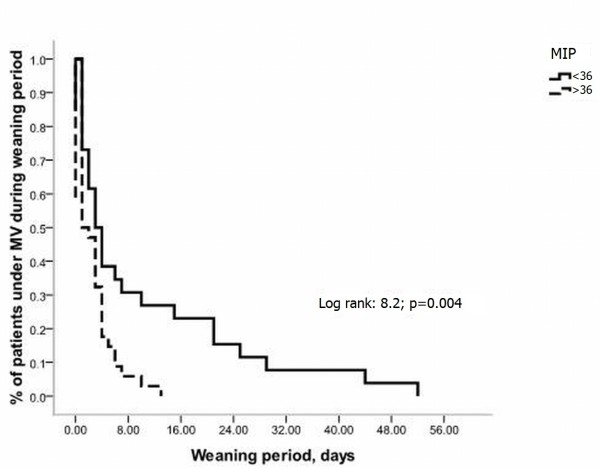
**Kaplan-Meier curves of the probability of remaining under mechanical ventilation (MV) after the onset of weaning according to maximum inspiratory pressure (MIP < or ≥ 36 cmH_2_O)**.

Similarly, patients with MIP < 36 cmH2O had a greater daily probability of remaining under mechanical ventilation (MV) after the onset of weaning compared with those patients with MIP ≥ 36 cmH2O (log-rank:8.2; p = 0.004, Figure 3).

## Discussion

Our study showed that MIP in critically ill patients significantly correlated with the MRC muscle strength score which estimates peripheral muscle strength.

Generalized, peripheral neuromyopathies can affect respiratory muscles and reduce respiratory pressures [18]. Chronic heart failure is a syndrome in which peripheral myopathy is characterized by skeletal muscle dysfunction, including the respiratory muscles [23,24]. There is a relationship between respiratory muscle strength and the oxidative capacity of the skeletal muscles in heart failure [25], indicating a correlation between the peripheral and the respiratory muscles.

De Jonghe et al [17] measured maximum inspiratory and expiratory pressures in critically ill patients on the first day of return to normal consciousness. The same day MRC muscle strength score was assessed. They discovered frequent alterations in the above mentioned variables of respiratory neuromuscular function. They also found a significant correlation between limb and respiratory muscle strength possibly suggesting a common basic pathophysiologic process which results in the noted limb and respiratory muscle weakness.

In our study we found a stronger correlation between MIP and MRC muscle strength score (r = 0.68 in our study vs r = 0.35 in the afore-mentioned study). Anxiety and inadequate collaboration up on the perception of the discontinued ventilatory support and/or the different method for MIP measurement might partly explain the difference. MIP value depends, among other factors, upon the lung volume when inspiratory effort begins. The use of the unidirectional valve (as in our study) permits MIP assessment at lung volumes progressively closer to residual volume, so that the patient can generate the greatest negative force while inspiring against the occluded airway and the measurement of maximal inspiratory muscle strength can be attained. This technique yields more reproducible, consecutive measurements and discloses values probably nearer the real MIP [26,27]. Its larger values could lessen the coefficient of variation.

In our study it was also demonstrated that MIP can predict weaning period from MV in the ICU, even in patients that could not cooperate. Both patient groups, with ICU-AW classified by the MRC score for muscle strength and patients with MIP below the cut-off point of 36 cmH_2_O, exhibited similarly prolonged weaning from the ventilator, supporting further the clinical relevance of MIP measurement in the critically ill, in regard to the ICU-AW.

Previous studies established a strong association between limb neuromuscular involvement and duration of mechanical ventilation. Addressing the question of whether ICU-AW prolongs mechanical ventilation and weaning time or if this prolongation is the effect of concurrent risk factors of weaning failure Garnacho-Montero et al [6], demonstrated that ICU-AW is an independent cause of weaning failure. Direct impact of ICU-AW on weaning duration was also previously demonstrated by De Jonghe et al [28]. In this study respiratory muscle weakness was associated with delayed extubation and prolonged ventilation [17]. Unlike the above mentioned study, in our study respiratory muscle assessment with MIP was also performed in patients that did not cooperate, providing information about weaning period and length of MV in this group of patients.

It has been shown that MIP can accurately be recorded in poorly cooperative patients when a unidirectional valve is used to occlude the airway, provided adequate respiratory drive is achieved [29]. In our study this method exhibited a very good ability to correctly classify those with and without ICU-AW.

Current approaches for ICU-AW diagnoses comprise physical motor examination and electrophysiological investigations. Physical examination is dependent on patient's cooperation and maximal effort. A reliable assessment of neuromuscular function (e.g. with MRC score determination) cannot be performed in patients not fully awake or when cognitive function is not intact.

Under these circumstances ICU-AW diagnosis, or generally neuromuscular dysfunction evaluation, relies on electrophysiological studies (motor and sensory nerve conduction studies, electromyography) [8,30-34] and electrical or magnetic nerve stimulation to measure isotonic or isometric limb muscle strength [35]. Moreover conventional signs of ICU-AW may be absent and electrophysiological studies could help establish the diagnosis. However, several limitations exist and also risks and costs of this testing should be taken into account. Conventional electrodiagnostic techniques often provide non-specific results or are hampered by local conditions (edema, multiple electrical devices) that prevent adequate disease classification [36]. Additionally, literature results are controversial regarding the estimation of ICU stay by electrophysiological studies. Witt et al found a negative correlation between the time of ICU stay and the peripheral nerve function as assessed by electrophysiological studies [33], whereas, in a more recent study, neurophysiologic testing results failed to predict duration of ICU stay or ventilation; it looks that they merely duplicate the diagnosis with no improvement to management or outcome prediction [37]. Advanced and invasive diagnostic procedures that require expertise in testing performance and interpretation are usually applied when patients exhibit not easily explained focal deficits or persistently altered sensorium. Therefore physical examination has been advocated as a primary determinant of ICU-AW [2].

Our study showed that MIP may be useful for peripheral muscle strength evaluation and ICU-AW identification in the critically ill, supporting its application in diagnostic decision making even when patients are unable to cooperate. It could be possibly considered as a screening test for ICU-AW as it can be performed earlier and in patients not fully awake, unlike MRC.

Several clinical implications exist. A reliable identification of this potentially reversible complication leads to most accurate predictions about a patient with protracted critical illness, which could alter the intensity of management [38]. Additionally early detection of the disease may force physicians and generally health providers to more consistently avoid precipitating factors. A recent study held in our institution addressed that electro muscular stimulation of muscles of lower extremities prevents the development of ICU-AW in critically ill patients and also results in shorter duration of weaning [39], indicating that therapeutic interventions could be applied earlier, possibly influencing outcome in terms of length of hospitalization, long term prognosis and mortality which are aggravated by the presence of ICU-AW.

## Limitations

A limitation of our study is the limited number of patients that eventually regained consciousness and were evaluated by both the MRC scale and MIP. The lack of an electrophysiological investigation is a limitation of the study and should be kept in mind when interpreting the significance of the results of this study. However we suggested MIP as a surrogate parameter of MRC muscle strength scale that is also a clinical approach for the detection of ICU-AW. Another limitation is that the results of the present study, specifically the ROC curve analysis and the AUC, which disclose the diagnostic accuracy of the utilized method, are based on the MIP and MRC score estimation in the selected group of patients that finally recovered, which questions the broader application of this method in all ICU patients (meaning in the non-recovering group). As there is not though so far a gold standard method for clinically relevant ICU-AW discrimination other than the physical assessment that requires patient cooperation and maximal effort, this is a general problem one encounters while attempting a non-invasive evaluation of patients with permanently reduced consciousness and comprehension. Finally, although we did not assess ventilatory drive before MIP assessment, forcing patients to boost a possibly insufficient ventilatory drive (add dead space for instance) would have resulted in even greater MIP values.

## Conclusions

In conclusion, measurement of MIP, utilizing the unidirectional valve method, in critically ill patients, may be used as a diagnostic tool for the early detection of ICU-AW even in patients that have not regained consciousness. Further investigations addressing the broad application of the method are required.

## List of abbreviations

APACHE: Acute Physiology and Chronic Health Evaluation; AUC: Area under the curve; ICU-AW: Intensive care unit acquired weakness; MIP: Maximum inspiratory pressure; MRC: Medical Research Council; MV: Mechanical ventilation; ROC: Receiver operating characteristic; SAPS: Simplified Acute Physiology score; SOFA: Sequential Organ Failure Assessment.

## Competing interests

The authors declare that they have no competing interests.

## Authors' contributions

All authors have contributed substantially to the submitted work and have read and approved the final manuscript. In particular GT participated in the design of the study, data acquisition, analysis, drafting of the manuscript and critically revised the manuscript. IV, DZ and SD participated in the design of the study, data analysis, drafting of the manuscript and critically revised the manuscript. EK, TP and ET participated in data acquisition, analysis and critically revised the manuscript. VG participated in the design of the study, data acquisition, analysis and critically revised the manuscript. CR participated in the design of the study and critically revised the manuscript. Finally, SN conceived of and helped with the coordination of the study, critically revised the manuscript and provided final approval.

## Pre-publication history

The pre-publication history for this paper can be accessed here:

http://www.biomedcentral.com/1471-2253/11/14/prepub
